# Exploring an anomaly: the synthesis of 7,7′-diazaindirubin through a 7-azaindoxyl intermediate†

**DOI:** 10.1039/d0ra07144g

**Published:** 2020-10-06

**Authors:** James A. Shriver, Katelyn R. Wang, Andrew C. Patterson, James R. DeYoung, Richard J. Lipsius

**Affiliations:** Central College 812 University St. Pella IA 50219 USA shriverj@central.edu

## Abstract

Two independent methods generating 7-azaindoxyl as an intermediate verify that 7,7′-diazaindirubin is formed exclusively over 7,7′-diazaindigo. This contrasts with long-standing knowledge related to the reactivity of indoxyl, which proceeds *via* a radical-initiated homodimerization process, leading to indigo. A series of experiments confirms 7-azaindoxyl as an intermediate with results suggesting a condensation pathway followed by oxidation.

## Abstract 2

Generation of 7-azaindoxyl under acidic conditions leads exclusively to 7,7′-diazaindirubin over 7,7′-diazaindigo through a condensation pathway.

## Graphical abstract

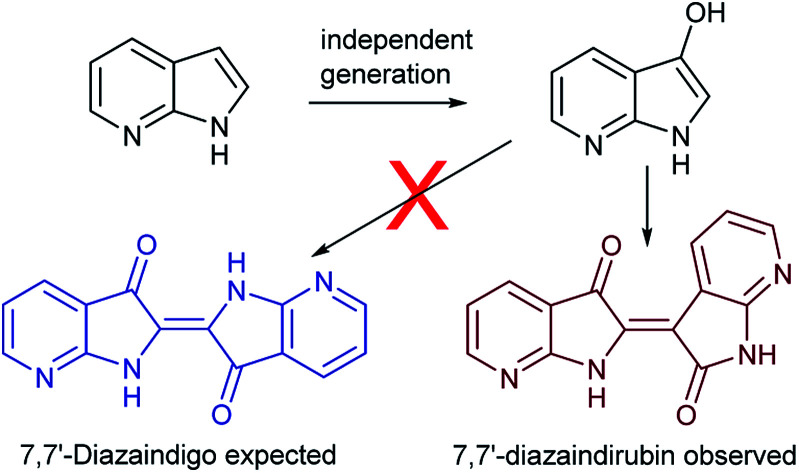

## Introduction

**Fig. 1:**
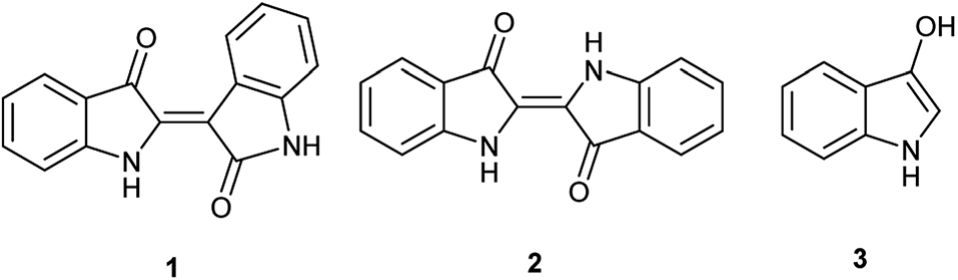
Chemical structures for indirubin (1), indigo (2) and indoxyl (3).

Indirubin (1), a side product from commercial indigo (2) production (Fig. 1), is emerging as a promising pharmaceutical platform. Indirubin derivatives have been studied for their anti-cancer properties,^1^ potential to treat diabetes,^2^ and anti-inflammatory response.^3^ Modern understanding began when the crystal structures, illustrating the interaction between cyclin dependent kinase-2 with both indirubin-3′-oxime and indirubin-5-sulfonate, were elucidated in 1999.^4^ Ultimately, this has led to promising leads *inter alia* in the treatment of gliomas,^5^ MLL leukemia cells,^6^ and pancreatic cancer for indirubin derivatives,^7^ and melanoma and non-small cell lung cancer cells for 7,7′-diazaindirubin derivatives.^8^

Indigo has been used and independently developed as a dye in many cultures,^9^ dated as early as 6000 years ago at an archaeological site in Peru.^10^ Traditionally, precursors are derived from natural sources that can vary with geographic location.^11^ These compounds give rise to indoxyl (3-hydroxyindole, 3, Fig. 1), a reactive intermediate that spontaneously converts to indigo in air with selectivity over indirubin. This has been confirmed through numerous studies of indoxyl-generating compounds under oxidative conditions, and it proceeds through a free radical-based mechanism. For example, the action by oxygen under basic conditions suggests a free radical process after generation of indoxyl.^12^ Moreover, convincing evidence^13^ by Berlin and co-workers supports this observation more broadly with other oxidants and includes the possibility of formation under neutral and acidic conditions with the appropriate oxidant.

**Scheme 1:**
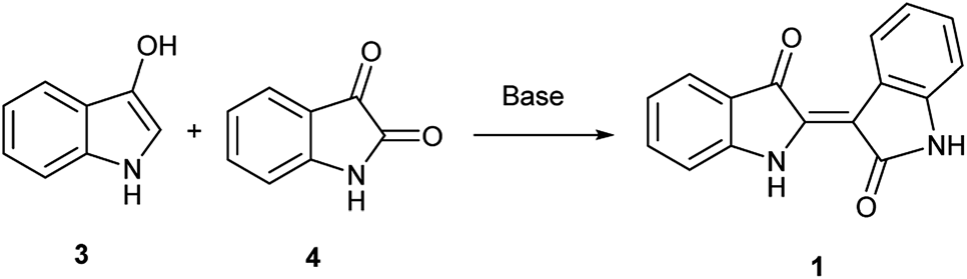
Original Baeyer synthesis of 1.

In contrast, the synthesis of indirubin is thought to occur due to formation of small amounts of isatin, 4, by oxidation of 3.^14^ Subsequent condensation occurs between indoxyl and isatin as leveraged in the traditional, and still dominant preparative method for indirubin introduced by Baeyer in the 1880s and illustrated in Scheme 1.^15^ Contemporaneously, there are a limited number of new preparations for indirubin through classical synthetic methods other than those which employ the classical Baeyer approach. In one example, 3-formyl indigo is reacted with Oxone® to form indirubin in a 6% yield as a biproduct during the synthesis of isatoic anhydrides.^16^ Though limited in its assessment, 4 was reacted with 2-oxoindole with PCl_5_ to form 5′ and 6′-substituted indirubins.^17^ A more promising protocol was accomplished through the reductive coupling of isatin using potassium borohydride^18^ where yields ranging from 63%–91% were seen for an array of electron-rich indirubins.

More recently, methods employing biological or enzyme-promoted pathways have been explored to generate indirubins and other indigoid species. Insight into this category of reaction can be gleaned by a look at traditional biological synthesis of indigo, which is nearly always accompanied by indirubin. An elaborated example of this is seen through the enzymatic hydrolysis of indican (indoxyl-β-d-glucoside) isolated from *Polygonum tincturum*.^19^ A tissue culture where *Polygonum tincturum* was fed with an array of indoles to produce indigo and indirubin without isolation.^20^ Additionally, Maugard identified isatan C during an early (June) harvest of woad, an indigo-producing plant, and discerned that it promoted increased formation of indirubin through an isatin precursor.^21^ A novel approach using Cytochrome P450 enzymes was highlighted in work by Guengerich which also noted an array of oxidation products beyond indoxyl including oxindole and dioxindole.^22^ Further elaboration of this process with Cytochrome P450 mutant enzymes formed an array of synthetic indirubin and indigo compounds.^23^ Recently, it was shown that cysteine can shift the selectivity of a flavin-containing monooxygenase from indigo to indirubin.^24^

**Fig. 2:**
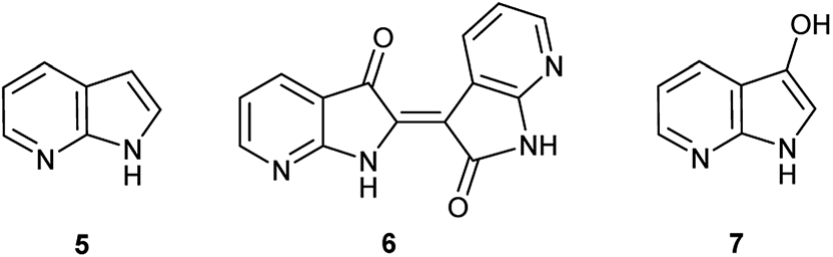
Chemical structures for 7-azaindole (5), 7,7′diazaindirubin (6), and 7-azaindoxyl (7).

Herein, an anomaly to the innate selectivity towards indigo under indoxyl-generating conditions is explored for 7-azaindole, 5, which exclusively produces 7,7′-diazaindirubin, 6. In this instance, the intermediate is specifically 7-azaindoxyl, 7 (Fig. 2). This preference was observed for two independent approaches, both of which show significant promise as viable platforms for the low-cost production of a wide array of indirubin derivatives.

## Results

**Scheme 2:**
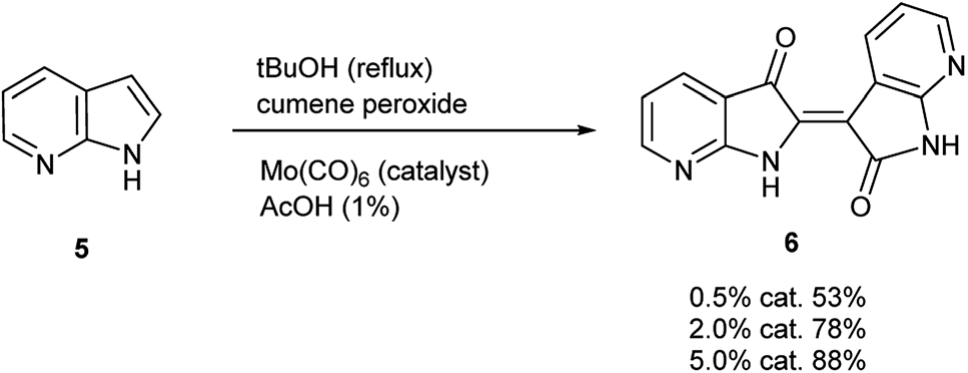
Molybdenum-catalyzed synthesis of 7,7′-diazaindirubin (6).

To initiate indirubin synthesis, a protocol developed by Yamamoto and co-workers was employed. Their work produced indigo from indole in up to 82% yield with a molybdenum catalyst in the presence of cumene peroxide.^25^ Utilizing a scaled-down variant of this procedure with increased catalyst load, this method converted 5 to 6 with no evidence of the corresponding indigo (Scheme 2).‡‡While initially scaled at 5 mmol, the reaction was performed once (non-optimized) at a 100 mmol scale to afford 5.66 g (43% yield) of 6.

**Scheme 3:**
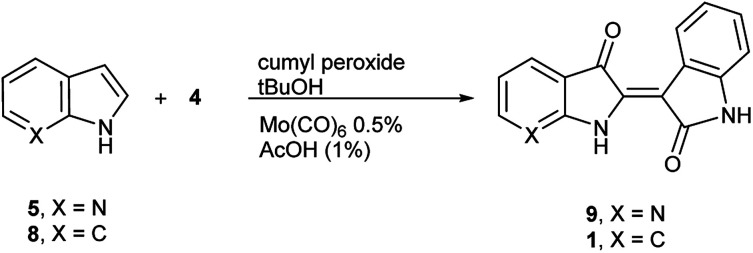
Trapping of indoxyl intermediates for molybdenum-catalysed procedure.

The next task was to confirm that the corresponding indoxyl was produced as an intermediate during the reaction. Germane to this goal, the original publication by Yamamoto *et al.* verified the presence of indoxyl through LCMS during the synthesis of 2. To independently test for the presence of an indoxyl intermediate under these conditions, 4 was employed as a trapping agent for indoxyl, harkening back to the original process developed by Baeyer shown in Scheme 1.^15^ For indole (8), indirubin (1) was produced preferentially over 2 in an 8 : 1 ratio. For 5, 7′-azaindirubin (9) was formed exclusively under standard reaction conditions (Scheme 3).

The protocol from Scheme 2 was applied to selected indoles to help assess the process. Broadly, the results illustrate that electron-deficient indoles are more amenable towards indirubin synthesis than relatively electron rich systems. Notably, 6-nitroindole, indole-5-carbonitrile and indole-5-carboxylic acid gave rise to appreciable quantities of the corresponding indirubin, while the parent system and 5-methoxyindole formed only indigo products. These results are summarized in Table 1, showing the percent recovery and relative abundance of indirubin and indigo, which have the same molecular weight, as assessed by ^1^H NMR integration.

**Table 1:** Relative formation of indirubin : indigo with 2% Mo(CO)_6_ catalyst

Indole	Indirubin	Indigo	Indirubin : indigo	Recovery%
Indole	NA	2	0 : 1	76%^a^
7-Aza	6	NA	1 : 0	78%
6-NO_2_	10	11	2 : 9	67%
5-CN	12	13	2 : 3	62%
5-CO_2_H	14	15	1 : 2	59%
5-OMe	NA	16	0 : 1	65%

a Yield of 82% obtained from Yamamoto *et al.*^25^

The second method to generate 6 was accomplished through the synthesis of 7-azaindoxyl acetate (17) and subsequent solvolysis of the acetate group. Synthesis of 17 was performed by oxidation of 5 with (diacetoxyiodo)benzene (DIB) using a modified protocol at larger scale (20 mmol), detailed in the ESI.†^26^ While saponification of indoxyl acetate is well known to produce indigo, mimicking reaction conditions under neutral-to-acidic conditions is more problematic. Ultimately, acid-catalysed transesterification§§The reaction was performed for 24 h with either trichloroacetic acid (14%) or Yb(OTf)_3_ (34%) to yield solely 6 without further optimization. at 150 °C gave rise to 6 as the sole product as shown in Scheme 3. In contrast, saponification of 17 leads to the corresponding indigo.^8^

**Fig. 3:**
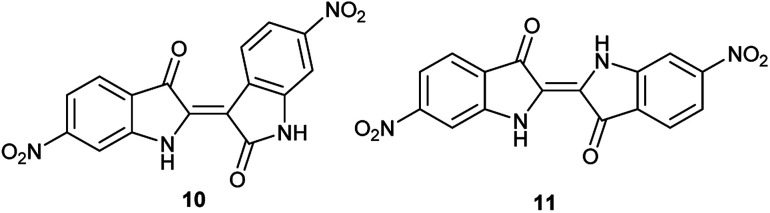
Structures of 6,6′-dinitroindirubin (10) and 6,6′-dinitroindigo (11).

**Fig. 4:**
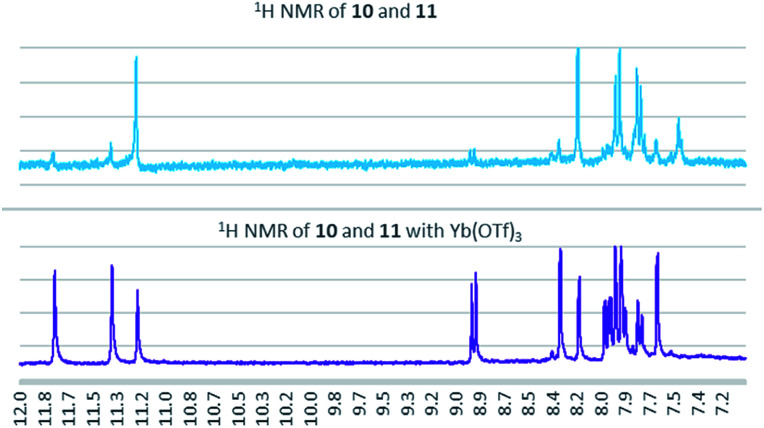
^1^H NMR for the formation of 10 and 11 with and without 2% Yb(OTf)_3_.

As a condensation pathway was suspected, a Lewis acid was used to facilitate indirubin formation. It has been established that rare earth triflates as Lewis acids are helpful in facilitating condensation reactions, including aldol condensations.^27^ The initial screening of indoles shown in Table 1 have generally produced a mixture of indigo and indirubin derivatives for electron deficient systems. The example of 6-nitroindole produced a preferred amount of 11 compared to 10 in a 2 : 9 ratio of 10 : 11 (Fig. 3). When 2% Yb(OTf)_3_ was added to the reaction, a 3 : 1 ratio of 10 : 11 was observed by NMR (Fig. 4).¶¶Due to low solubility, the ^1^H NMR was taken with 2000 scans at 300 MHz on the crude precipitate after filtration. It is important to note that Yb(OTf)_3_ inhibited total recovery of product(s) for all examples explored, likely due to a deleterious effect on the molybdenum catalyst.

## Discussion

The collective results supply insight into the likely mechanism for this reaction. The reaction in Scheme 2 illustrated an unambiguous switch in selectivity from indigo to indirubin when utilizing 5 instead of indole as the reactant. It was initially assumed that this observation was due to the electron deficient character of 5, which was subsequently supported in the evaluation shown in Table 1 for other substituted indoles. As this reaction was already known to proceed through an indoxyl intermediate from prior research, this strongly suggests that the ability of the 3-position in indoxyl to behave as an electrophile is enhanced. To verify the results from Yamamoto for the parent system and to confirm that an indoxyl intermediate is also present for 5, a trapping experiment with 4 was run as shown in Scheme 3. This result helps substantiate that an alternate mechanism, with initial oxidation at the 2-position, is not in play.

**Scheme 4:**
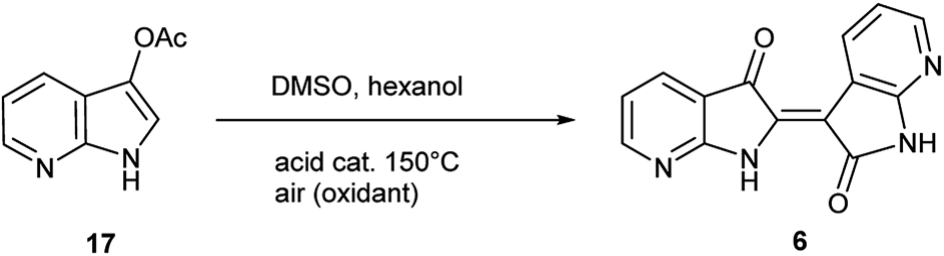
Acid-catalysed transesterification on 17 leading to 6.

**Scheme 5:**
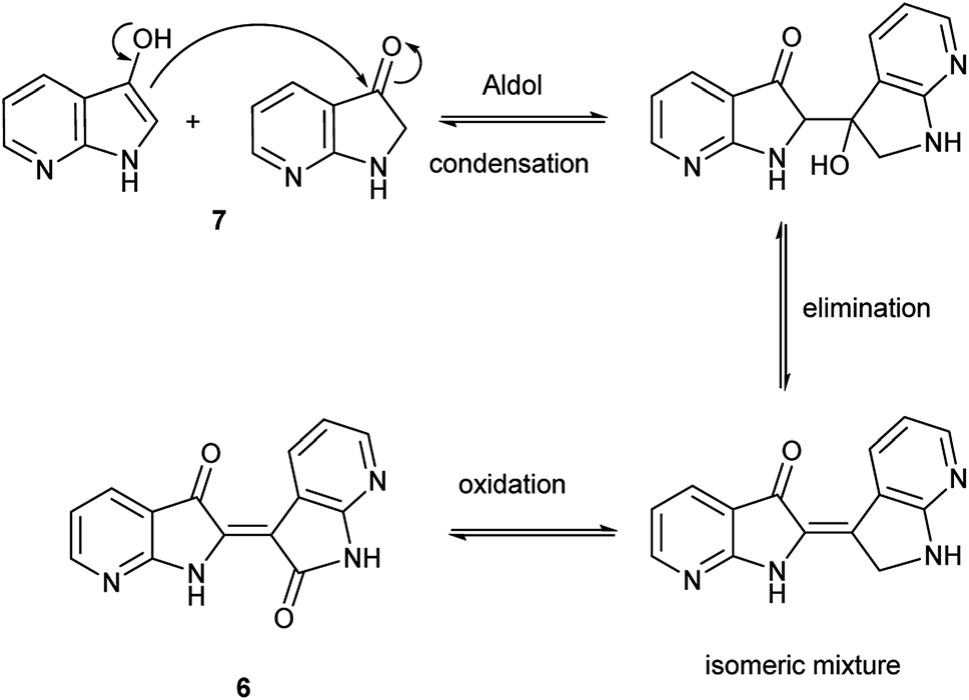
Proposed mechanism for the synthesis of 6 from 7.

Given the complexity of the system from Scheme 2, it was important to independently verify this result, allowing removal of peroxide and the molybdenum catalyst as variables. Scheme 4 illustrates this alternate approach, which lead to the same result under acidic (but not basic) conditions. Moreover, the fact that peroxide was not necessary to promote this transformation strongly suggests that the reaction is proceeding through condensation pathway to generate 7 and is followed by oxidation with air to form 6. This was further affirmed by the addition of a rare earth triflate, which promoted the formation of indirubin over indigo for 6-nitroindole. A mechanistic pathway is illustrated in Scheme 5, which hypothesizes the intermolecular aldol condensation between the keto and enol forms of 7 followed by oxidation with air (or peroxide).

## Conclusions

In summary, the anomalous behaviour where certain indoxyl compounds form indirubin, illustrated in detail for 7, is likely due to a previously unidentified condensation pathway shown in Scheme 4. This is a striking result given the ubiquitous nature of indigo and the extent of research around it. Moreover, there is significant opportunity for the scope to include, at the least, an array of electron deficient indoles. This is particularly true given the indirubin-enhancing effect seen for Yb(OTf)_3_. In addition to possible methodology development, future studies will explore the extent of keto–enol tautomerization and its role in promoting this anomalous pathway.

## Conflicts of interest

There are no conflicts to declare.

## Supplementary Material

RA-010-D0RA07144G-s001
