# Supplementary material for: Exploring an anomaly: the synthesis of 7,7′-diazaindirubin through a 7-azaindoxyl intermediate
Source: RSC Adv. 2020 Oct 6;10(60):36849–52. doi: 10.1039/d0ra07144g (PMC9057083; doi:10.1039/d0ra07144g)
Supplement: RA-010-D0RA07144G-s001 [file RA-010-D0RA07144G-s001.pdf]

Electronic Supplementary Material (ESI) for RSC Advances.
This journal is © The Royal Society of Chemistry 2020

Supporting Information

Exploring an Anomaly: The Synthesis of 7,7'-Diazaindirubin through a 7-Azaindoxyl Intermediate

James A. Shriver, Katelyn R. Wang, Andrew C. Patterson, James R. DeYoung, Richard J. Lipsius

Correspondence may be to directed to:

James A. Shriver
Central College
Pella, IA 50219
shriverj@central.edu

Table of Contents

1. General Methods and Instrumentation……………………………………………………………………….S-2

2. Synthetic Details………………………………………………………………………………………………………S-3

3. NMR Spectra……S-6

4. References………S-18

1. General Methods and Safety

All starting materials were commercially available and used without additional purification from TCI, Aldrich, Fisher Scientific and Alfa Aesar. All NMR spectra were determined in d^6-DMSO at 300 MHz (^1H NMR) on a Bruker Avance™ spectrometer. The chemical shifts are expressed in δ values relative to the appropriate solvent. For reaction mixtures, NMR integration was used to assess approximate relative values using high scan number to optimize accuracy.

2. Synthetic Details.

7,7'-Diazaindirubin (**6**)

Method 1: To a round bottomed flask, 0.591 g (5 mmol) of 7-azaindole, 0.066 g (0.25 mmol) of $Mo(CO)_6$, 1.63 mL (11 mmol) of an 80% solution of cumyl hydroperoxide in cumene, 0.015 mL (0.25 mmol) of acetic acid and 10.9 mL of t-butanol were added. The round bottomed flask was then placed in an oil bath set to 86°C. After 24 hours the product was put in an ice bath for 20 minutes, vacuum filtered, washed with cold methanol and placed on a high vacuum pump for 3 hours to afford a brick red solid (0.580 g, 2.19 mmol, 87.8% yield).

Method 2: To a round bottomed flask 0.176 g (1 mmol) of 7-azaindoxyl acetate (**6**), 0.012 g (0.02 mmol) of ytterbium (III) triflate, 4 mL of DMSO and 1 mL of hexanol were added. The round bottomed flask was then placed in an oil bath set to 150°C. After 24 hours the product was put in an ice bath and 15 mL of water was added to force precipitation. The product was then vacuum filtered, washed with a 1:1 solution of cold methanol and water and placed on the high vacuum pump for 3 hours to afford a brick red solid (0.045 g, 0.170 mmol, 34.1% yield).

Spectroscopy: ^{1}H NMR of **2** was previously published[1] ^{1}H NMR (300 MHz, d_6-DMSO) δ- 11.60 (1H, s); 10.76 (1H, s); 8.86 (1H, d, *J* = 7.5 Hz); 8.52 (1H, dd, *J* = 5.1 Hz and 1.5 Hz); 8.13-8.18 (2H, m); 7.16 (1H, dd, 7.5 Hz and 5.1 Hz); 7.11 (1H, dd, *J* = 7.2 Hz and 5.4 Hz).

Indirubin (1)

To a round bottomed flask 0.588 g (5 mmol) of indole, 0.736 g (5 mmol) of isatin, 0.006 g (0.025 mmol) of $Mo(CO)_6$, 0.813 mL (5.5 mmol) of an 80% solution of cumyl hydroperoxide in cumene, 0.015 mL (0.25 mmol) of acetic acid and 10.9 mL of t-butanol were added. The round bottomed flask was then placed in an oil bath set to 55°C. After 24 hours the product was put in an ice bath for 20 minutes, vacuum filtered, washed with cold methanol and placed on a high vacuum pump for 3 hours to afford 0.673 g (2.57 mmol) of a dark purple solid. This was a 51.3% recovery with an indirubin to indigo product ratio of 8:1 as determined by an ^{1}H NMR.

Spectroscopy: ^{1}H NMR of **6** was previously published[2] ^{1}H NMR (300 MHz, d_6-DMSO) δ- 11.03 (1H, s); 10.91 (1H, s); 8.78 (1H, d, *J* = 7.5 Hz); 7.66 (1H, d, *J* = 7.2 Hz); 7.59 (1H, t, *J* = 7.5 Hz); 7.43 (1H, d, 8.1 Hz); 7.27 (1H, t, *J* = 7.4Hz); 7.01-7.07 (2H, m); 6.91 (1H, d, *J* = 7.8 Hz).

7'-Azaindirubin (9)

To a round bottomed flask 0.591 g (5 mmol) of 7-azaindole, 0.736 g (5 mmol) of isatin, 0.006 g (0.025 mmol) of molybdenumhexacarbonyl, 1.63 mL (11 mmol) of an 80% solution of cumyl hydroperoxide in cumene, 0.015 mL (0.25 mmol) of acetic acid and 10.9 mL of tert-butanol were added. The round bottomed flask was then placed in an oil bath set to 86°C. After 24 hours the product was put in an ice bath for 20 minutes, vacuum filtered, washed with cold methanol and placed on a high vacuum pump for 3 hours to afford a brick red solid (0.138 g, 0.522 mmol, 10.5% yield).

Spectroscopy: ^{1}H NMR of **7** was previously published[1] ^{1}H NMR (300 MHz, d_6-DMSO) δ- 11.05 (1H, s); 10.81 (1H, s); 8.71 (1H, d, *J* = 7.5 Hz); 8.51 (1H, d, *J* = 4.8 Hz); 8.13 (1H, d, *J* = 6.9 Hz); 7.32 (1H, t, 7.2 Hz); 7.05-7.15 (2H, m); 6.96 (1H, d, *J* = 7.2 Hz).

Indigo (2)

To a round bottomed flask 0.588 g (5 mmol) of indole, 0.0660 g (0.25 mmol) of $Mo(CO)_6$, 1.63 mL (11 mmol) of an 80% solution of cumyl hydroperoxide in cumene, 0.0143 mL (0.25 mmol) of acetic acid and 10.9 mL of tert-butanol were added. The round bottomed flask was then placed in an oil bath set to 86 °C. After 24 hours the product was put in an ice bath for 20 minutes, vacuum filtered, washed with cold

methanol and placed on a high vacuum pump for 3 hours to afford 0.517 g (1.97 mmol) of a dark blue/black solid in a 79% yield as affirmed by ^{1}H NMR.
Spectroscopy: ^{1}H NMR was consistent with widely available spectra.[3] ^{1}H NMR (300 MHz, d_6-DMSO) δ- 10.51 (2H, s); 7.62 (2H, d, *J* = 7.5 Hz); 7.52 (2H, t, J = 7.2 Hz); 7.34 (2H, d, J = 8.1 Hz); 6.96 (2H, t, J = 8.4 Hz).

6,6'-dinitroindirubin (10) and 6,6'-dinitroindigo (11)
Method 1: To a round bottomed flask 0.811 g (5 mmol) of 6-nitroindole, 0.0660 g (0.25 mmol) of $Mo(CO)_6$, 1.63 mL (11 mmol) of an 80% solution of cumyl hydroperoxide in cumene, 0.0143 mL (0.25 mmol) of acetic acid and 10.9 mL of tert-butanol were added. The round bottomed flask was then placed in an oil bath set to 86 °C. After 24 hours the product was put in an ice bath for 20 minutes, vacuum filtered, washed with cold methanol and placed on a high vacuum pump for 3 hours to afford 0.592 g (1.68 mmol) of a blue solid. This was a 67% recovery with a **10:11** ratio of 2:9 as determined by an ^{1}H NMR.
Method 2: To a round bottomed flask 0.811 g (5 mmol) of 6-nitroindole, 0.0264 g (0.1 mmol) of molybdenumhexacarbonyl, 1.63 mL (11 mmol) of an 80% solution of cumyl hydroperoxide in cumene, 0.0620 g (0.1 mmol) of ytterbium (III) triflate and 10.9 mL of tert-butanol were added. The round bottomed flask was then placed in an oil bath set to 86 °C. After 24 hours the product was put in an ice bath for 20 minutes, vacuum filtered, washed with cold methanol and placed on a high vacuum pump for 3 hours to afford 0.159 g (0.451 mmol) of a dark purple solid. This was a 18% recovery with a **10:11** ratio of 3:1 as determined by an ^{1}H NMR spectra recorded on a Bruker 300 MHz spectrometer.
Spectroscopy: ^{1}H NMR was previously published for **10** and **11**.[4] ^{1}H NMR for **10**, (300 MHz, d_6-DMSO) δ- 11.75 (1H, s); 11.36 (1H, s); 8.89 (1H, d, *J* = 8.7 Hz); 8.30 (1H, d, *J* = 2.1 Hz); 7.98 (1H, dd, *J* = 8.7 Hz and 2.1 Hz); 7.92-7.87 (2H, m); 7.64 (1H, d, *J* = 2.1 Hz). For **11** ^{1}H NMR (300 MHz, d_6-DMSO) δ- 11.20 (2H, s); 8.18 (2H, d, *J* = 1.8 Hz); 7.91 (2H, d, J = 8.1 Hz); 7.75 (2H, dd, J = 8.1Hz and 1.8Hz).

Indirubin- 5,5'-dicarbonitrile (12) and Indigo-5,5'-dicarbonitrile (13)
To a round bottomed flask 0.711 g (5 mmol) of 5-cyanoindole, 0.0660 g (0.25 mmol) of $Mo(CO)_6$, 1.63 mL (11 mmol) of an 80% solution of cumyl hydroperoxide in cumene, 0.0143 mL (0.25 mmol) of acetic acid and 10.9 mL of tert-butanol were added. The round bottomed flask was then placed in an oil bath set to 86 °C. After 24 hours the product was put in an ice bath for 20 minutes, vacuum filtered, washed with cold methanol and placed on a high vacuum pump for 3 hours to afford 0.483 g (1.55 mmol) of a purple solid. This was a 62% recovery with a **12:13** ratio of 2:3 as determined by an ^{1}H NMR.
Spectroscopy: ^{1}H NMR was previously published for **12** and **13**.[3] ^{1}H NMR for **12**, ^{1}H NMR (300 MHz, d_6-DMSO) δ- 11.57 (1H, s); 11.48 (1H, s); 9.05 (1H, d, *J* = 1.7 Hz); 8.21 (1H, d, *J* = 0.9 Hz); 8.01 (1H, dd, *J* = 8.4 Hz and 1.8 Hz); 7.75 (1H, dd, *J* = 8.4 Hz and 1.8 Hz) 7.60 (1H, d, *J* = 8.4 Hz). 7.08 (1H, d, *J* = 8.1 Hz). For **13** ^{1}H NMR (300 MHz, d_6-DMSO) δ- 11.20 (2H, s); 8.14 (2H, d, *J* = 1.2 Hz); 7.91 (2H, dd, J = 8.4 Hz and 1.5 Hz); 7.48 (2H, d, J = 8.1Hz).

Indirubin- 5,5'-dicarboxylic acid (14) and Indigo-5,5'-dicarboxylic acid (15)
To a round bottomed flask 0.711 g (5 mmol) of indole-5-carboxylic acid, 0.0660 g (0.25 mmol) of $Mo(CO)_6$, 1.63 mL (11 mmol) of an 80% solution of cumyl hydroperoxide in cumene, 0.0143 mL (0.25 mmol) of acetic acid and 10.9 mL of tert-butanol were added. The round bottomed flask was then placed in an oil bath set to 86 °C. After 24 hours the product was put in an ice bath for 20 minutes, vacuum filtered, washed with cold methanol and placed on a high vacuum pump for 3 hours to afford 0.512 g (1.46 mmol) of a purple solid. This was a 59% recovery with a **14:15** ratio of 1:2 as determined by an ^{1}H NMR. A small quantity of an unidentified impurity, likely an indigoid, was also present.

Spectroscopy: ^{1}H NMR was previously published for **14**[5] and **15**.[6] For **14**, numerous peaks were obscured by the larger peaks for **15**. For **15** ^{1}H NMR (300 MHz, d_6-DMSO) δ- 12.82 (2H, s); 11.03 (2H, s); 8.13 (2H, d, *J* = 1.5 Hz); 8.10 (2H, dd, J = 8.4 Hz and 1.5 Hz); 7.40 (2H, d, J = 8.4 Hz).

5,5'-Dimethoxyindigo(16)

To a round bottomed flask 0.736 g (5 mmol) of 5-methoxyindole, 0.0660 g (0.25 mmol) of $Mo(CO)_6$, 1.63 mL (11 mmol) of an 80% solution of cumyl hydroperoxide in cumene, 0.0143 mL (0.25 mmol) of acetic acid and 10.9 mL of tert-butanol were added. The round bottomed flask was then placed in an oil bath set to 86 °C. After 24 hours the product was put in an ice bath for 20 minutes, vacuum filtered, washed with cold methanol and placed on a high vacuum pump for 3 hours to afford 0.521 g (1.62 mmol) of a blue solid in a 65% yield.

Spectroscopy: ^{1}H NMR was not previously published for **16**, though it has been made on multiple occasions.[7] A clean ^{1}H NMR and ^{13}C NMR (40,000 scans) were obtained for characterization.

For **14** ^{1}H NMR (300 MHz, d_6-DMSO) δ- 10.27 (2H, s); 7.28 (2H, d, *J* = 9.0 Hz); 7.16 (2H, dd, J = 9.0 Hz and 2.7 Hz); 7.09 (2H, d, J = 2.4 Hz); 3.78 (6H, s). ^{13}C NMR (75 MHz, d_6-DMSO) δ-188.04; 154.21; 148.13; 125.26; 122.04; 119.55; 115.03; 105.27; 56.09.

7-azaindoxyl acetate (17)

In a modified synthetic procedure,[8] to a round bottomed flask 2.363 g (20 mmol) of 7-azaindole, 9.663 g (30 mmol) of diacetoxyiodobenzene, 2.244 g (40 mmol) of potassium hydroxide and 100 mL of acetonitrile were added. The round bottomed flask was then placed in an oil bath set to 50°C. After 24 hours the product was placed into a separatory funnel with 600 mL of water and 150 mL of dichloromethane. Once separated, the water layer was washed twice more with 150 mL of dichloromethane. The organic layer was then washed twice with 300 mL of water and finally with 300 mL of brine solution. Magnesium sulfate was added to the dichloromethane solution for to dry, the product was then vacuum filtered and placed on the rotary evaporator. A column was run using 1:1 hexane to ethyl acetate as the eluent. The product was then collected, placed on the rotary evaporator and the high vacuum pump for 3 hours to afford a light pink solid (1.163 g, 6.61 mmol, 33% yield).

Spectroscopy: The ^{1}H NMR did not match with the original published procedure, which looks to be incorrectly assigned or transcribed. An independent study using thallium acetate,[1] aligned with our NMR data. ^{1}H NMR (300 MHz, d_6-DMSO) δ- 11.57 (1H, s); 8.26 (1H, d, *J* = 3.3 Hz); 7.84 (1H, d, *J* = 7.5 Hz); 7.44 (1H, d, *J* =2.4 Hz); 7.09 (1H, dd, *J* = 7.8 and 4.8 Hz); 2.33 (3H, s).

3. NMR

^{1}H NMR of **6** (Method 1)

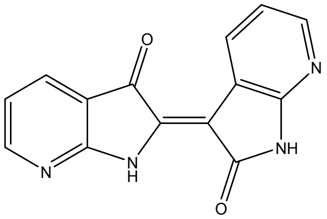

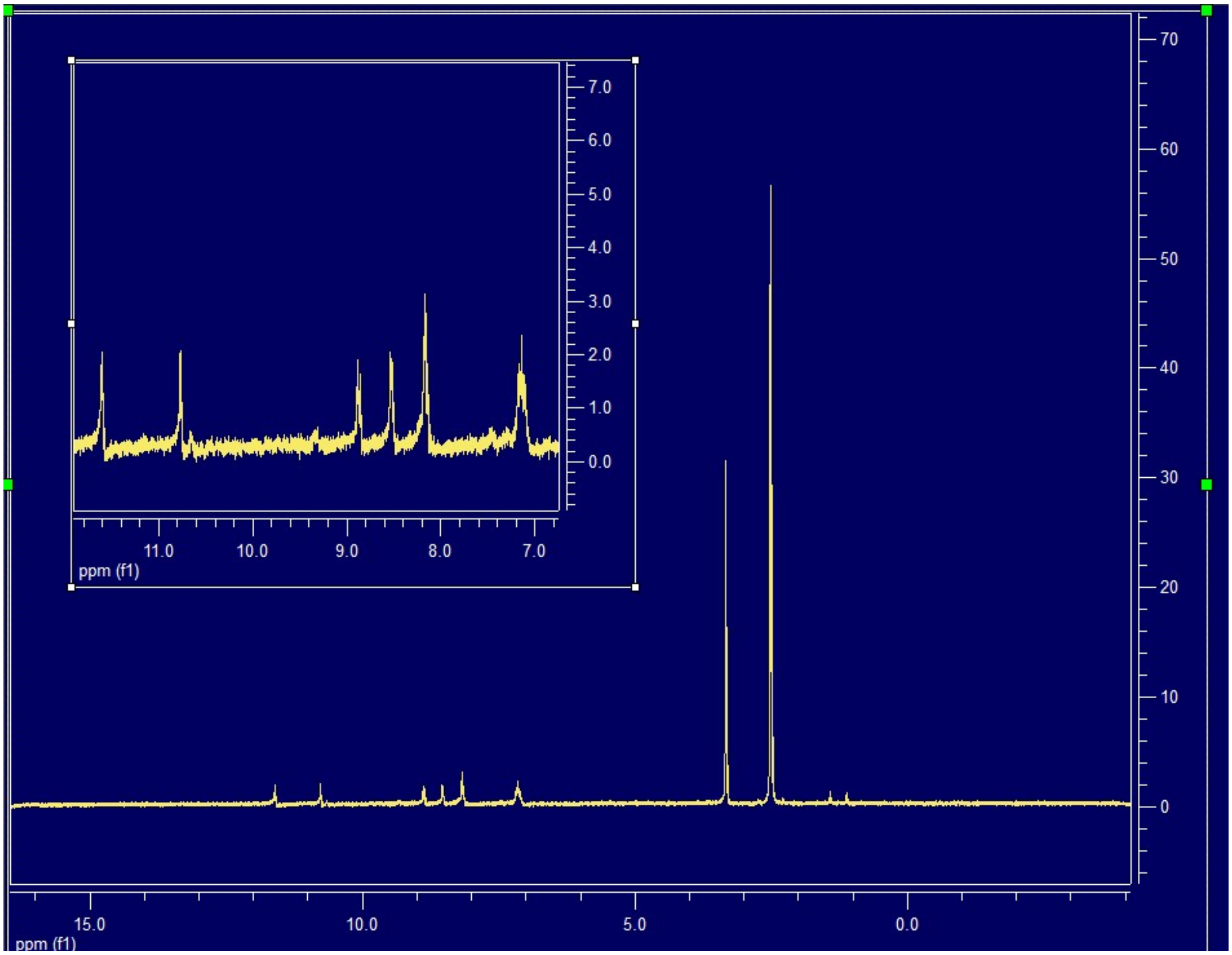

^{1}H NMR of **6** (Method 2)

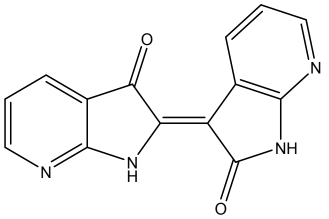

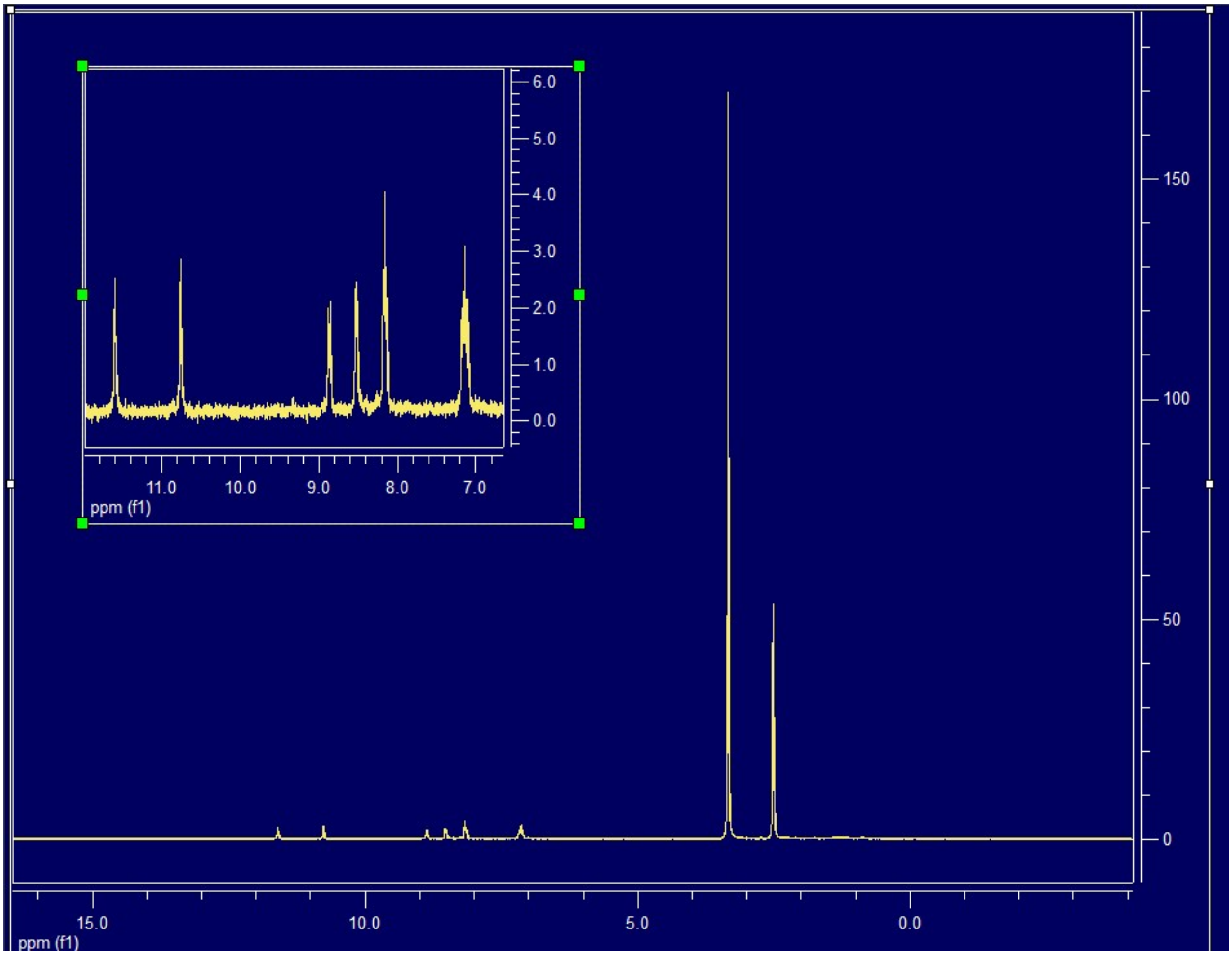

^{1}H NMR of **1** (with indole).

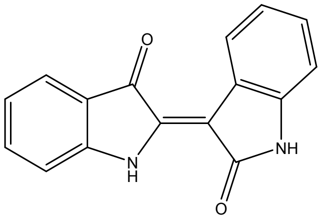

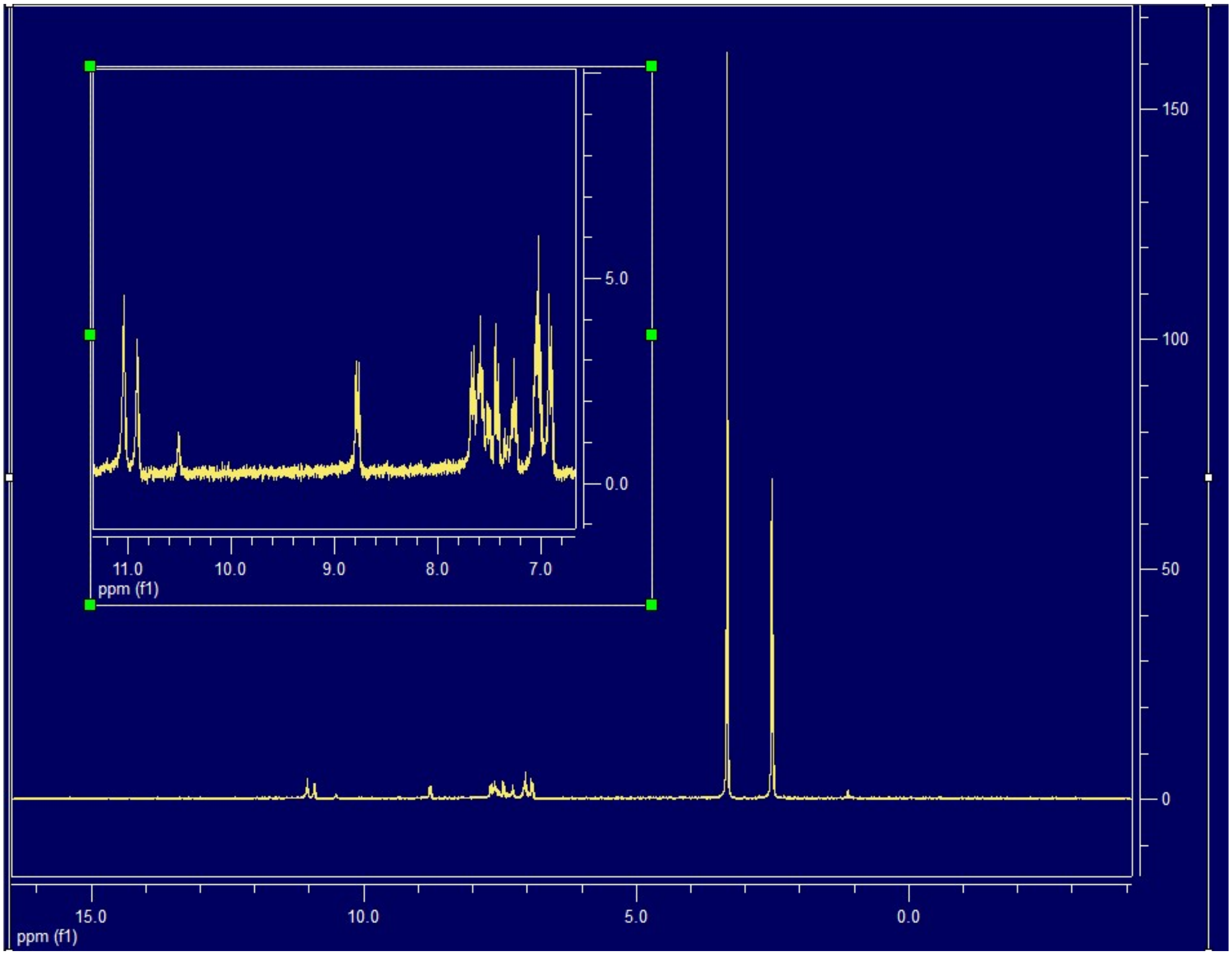

^{1}H NMR of **9**.

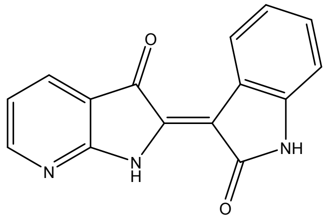

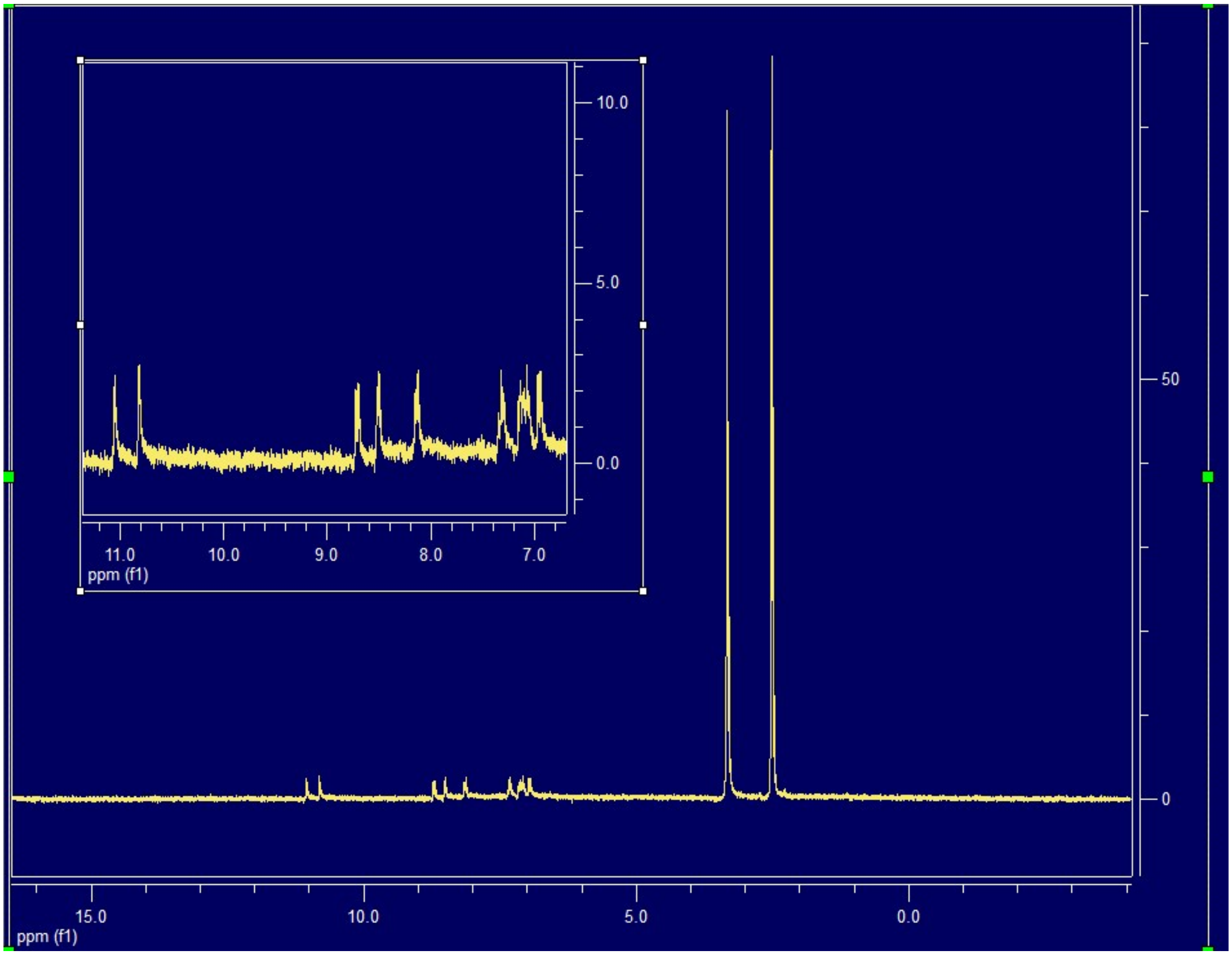

^{1}H NMR of **2**.

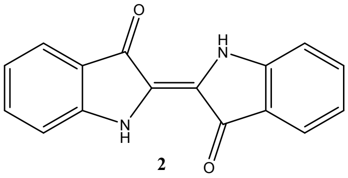

O
H
N
N
H
O

2

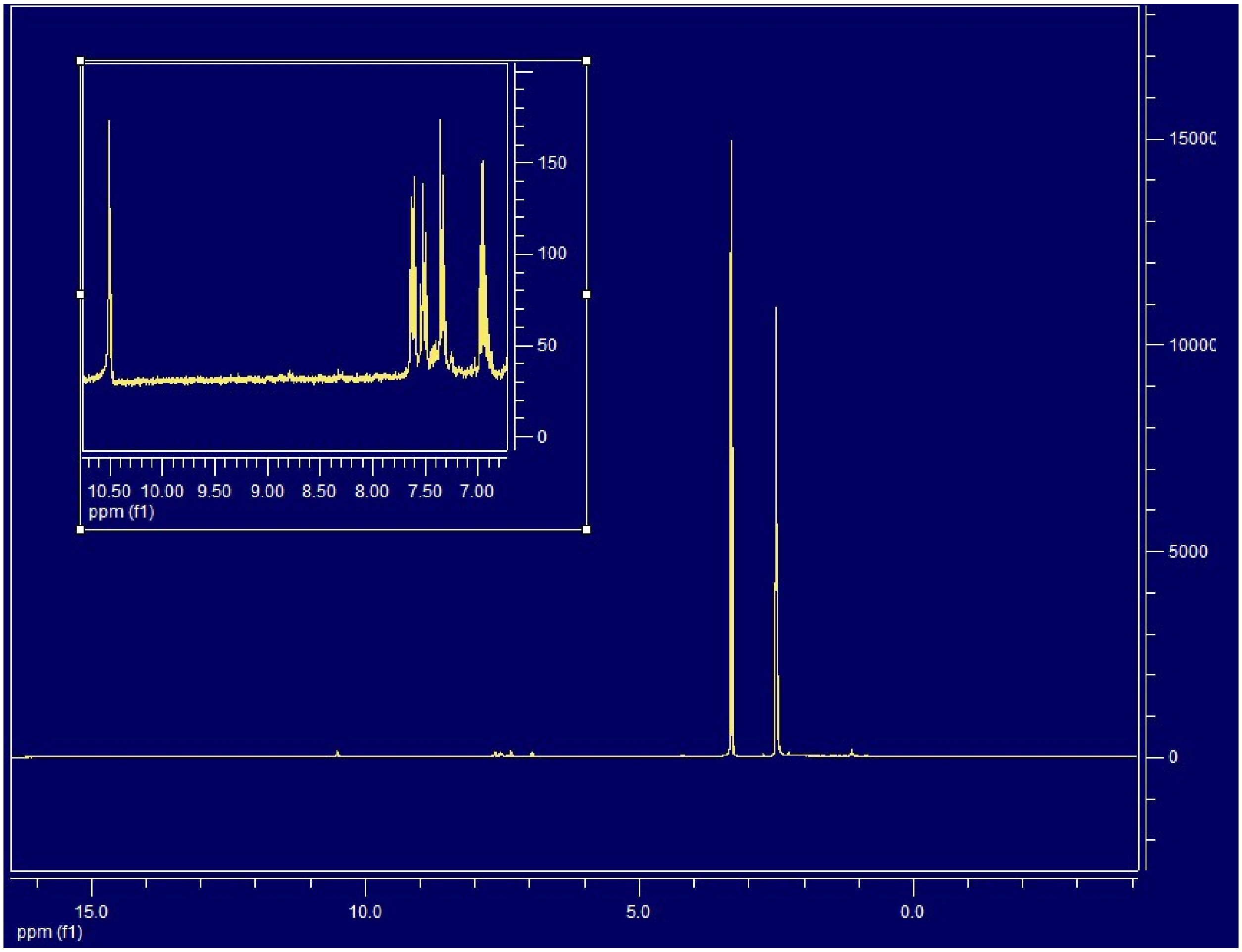

150
100
50
0
10.50 10.00 9.50 9.00 8.50 8.00 7.50 7.00
ppm (f1)
15000
10000
5000
0
15.0
10.0
5.0
0.0
ppm (f1)

^{1}H NMR of **10** and **11** no $Yb(OTf)_3$

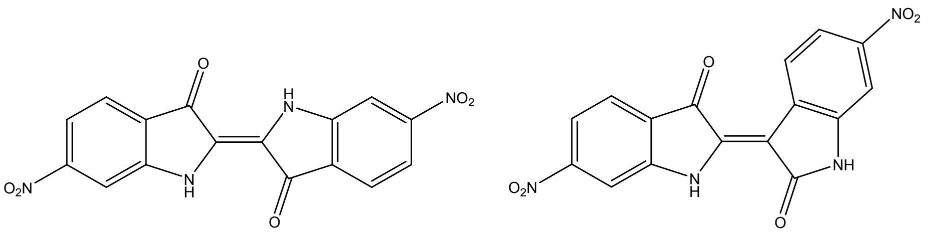

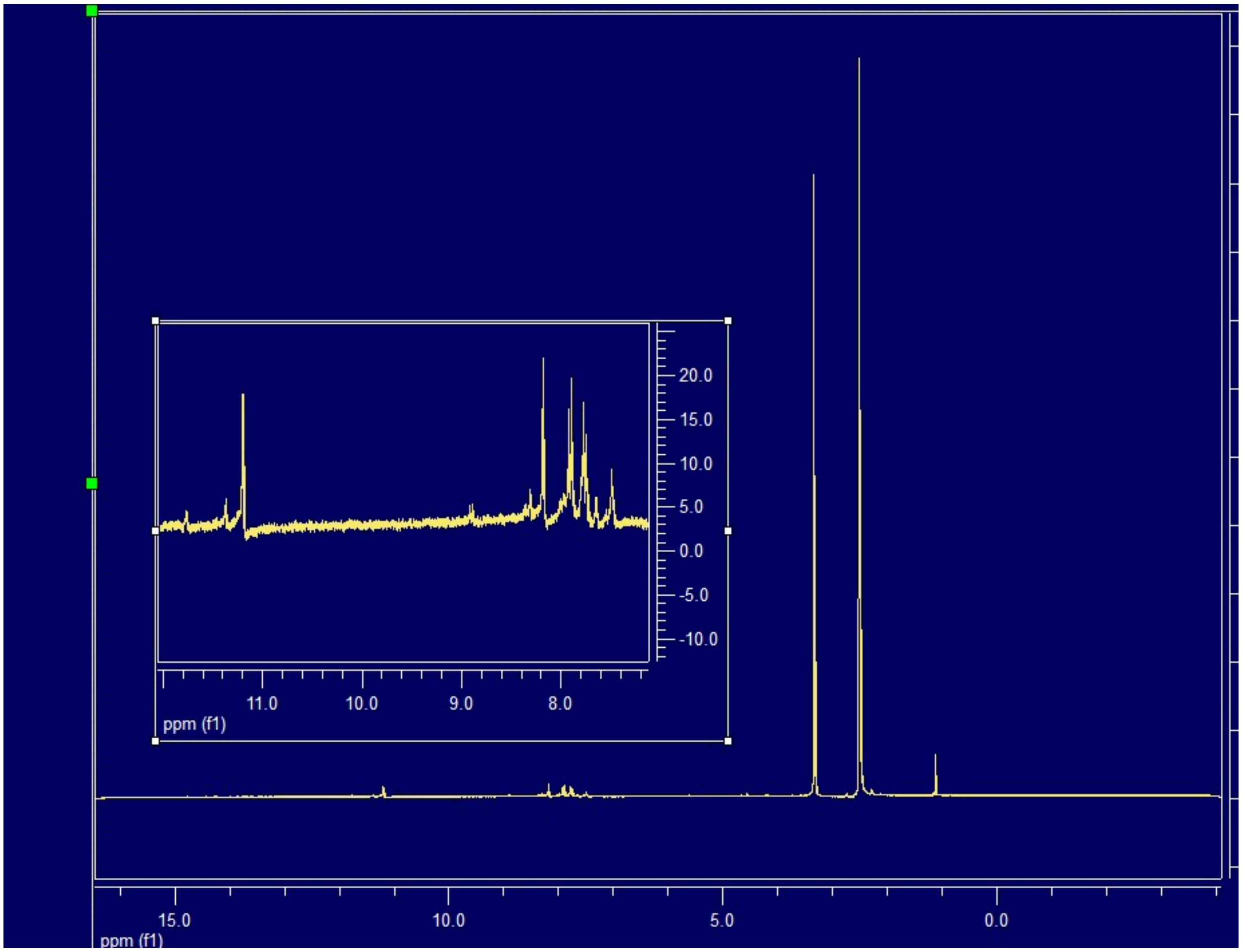

^{1}H NMR of **10** and **11** with $Yb(OTf)_3$

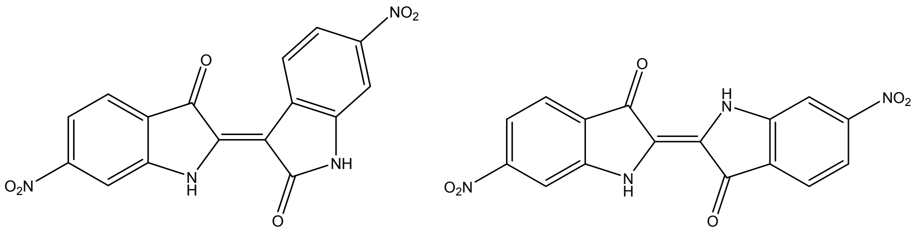

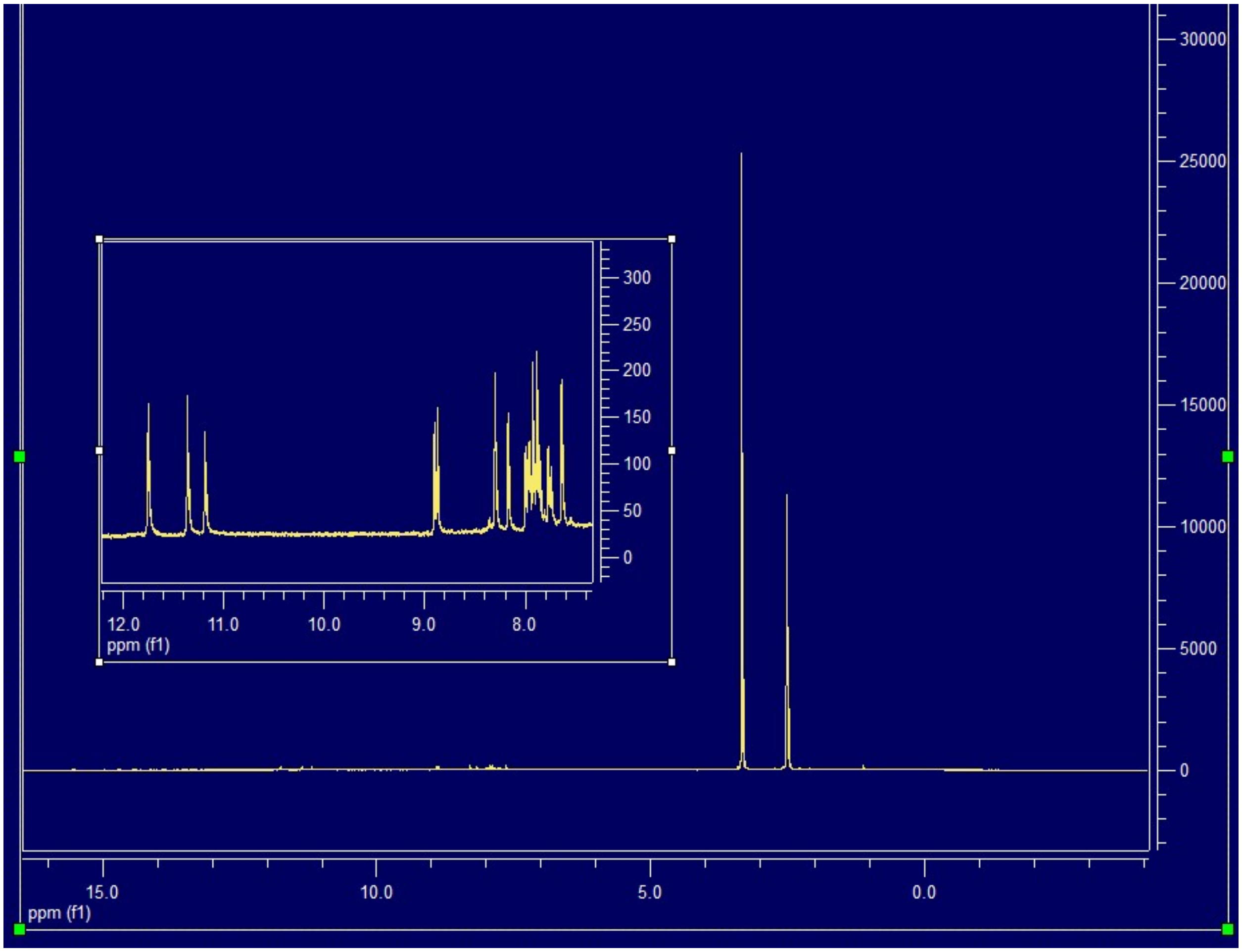

^{1}H NMR of **12** and **13.**

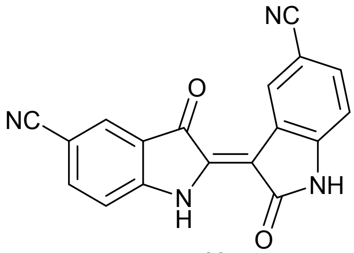

12

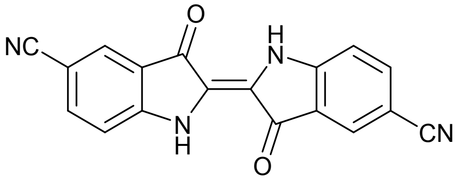

13

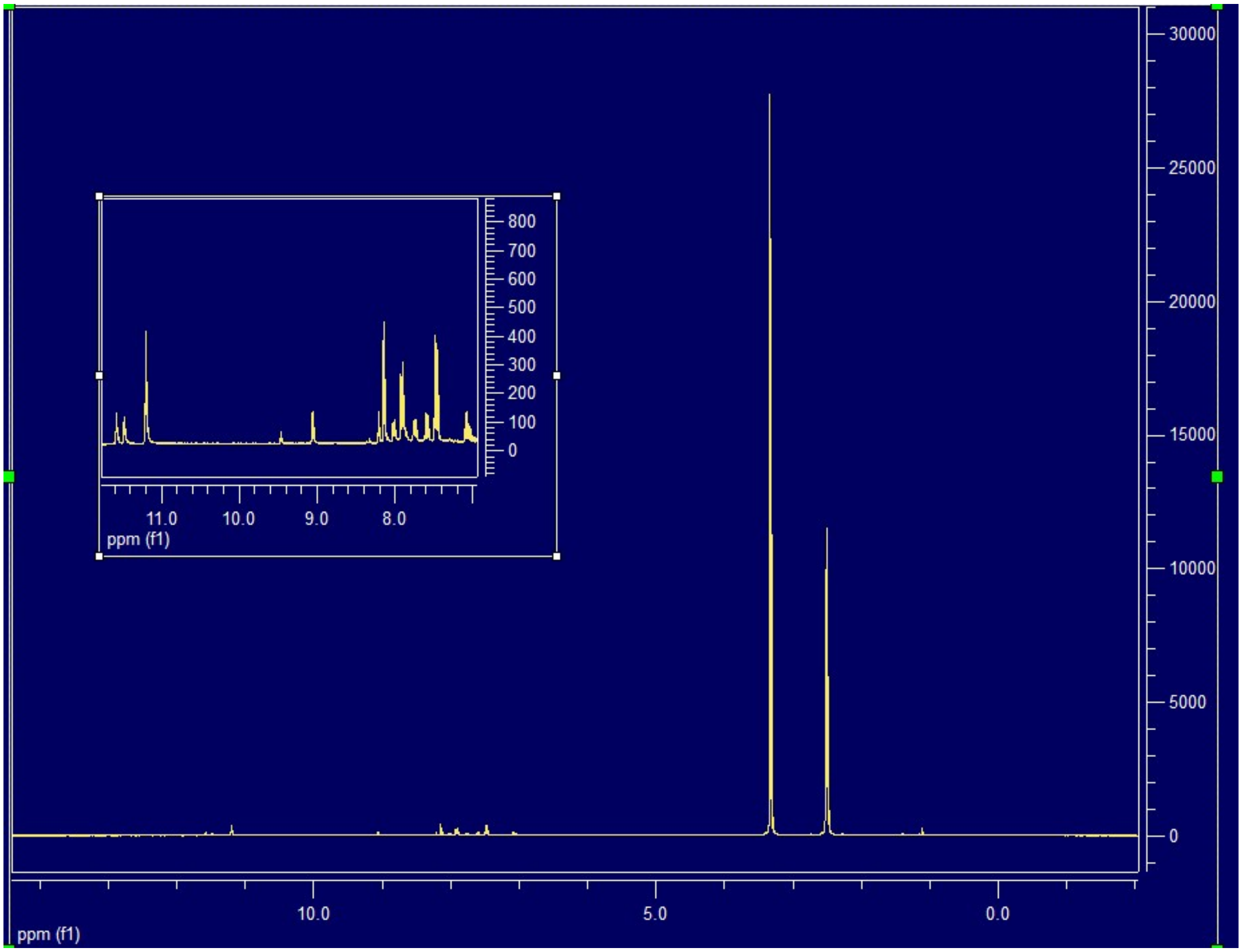

^{1}H NMR of **12** and **13.**

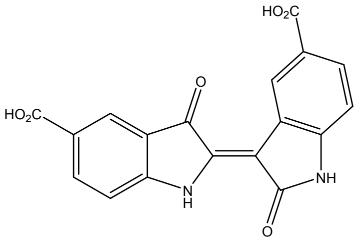

14

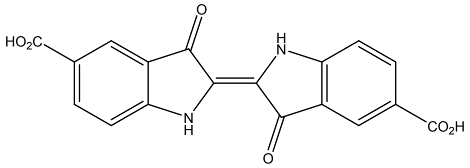

15

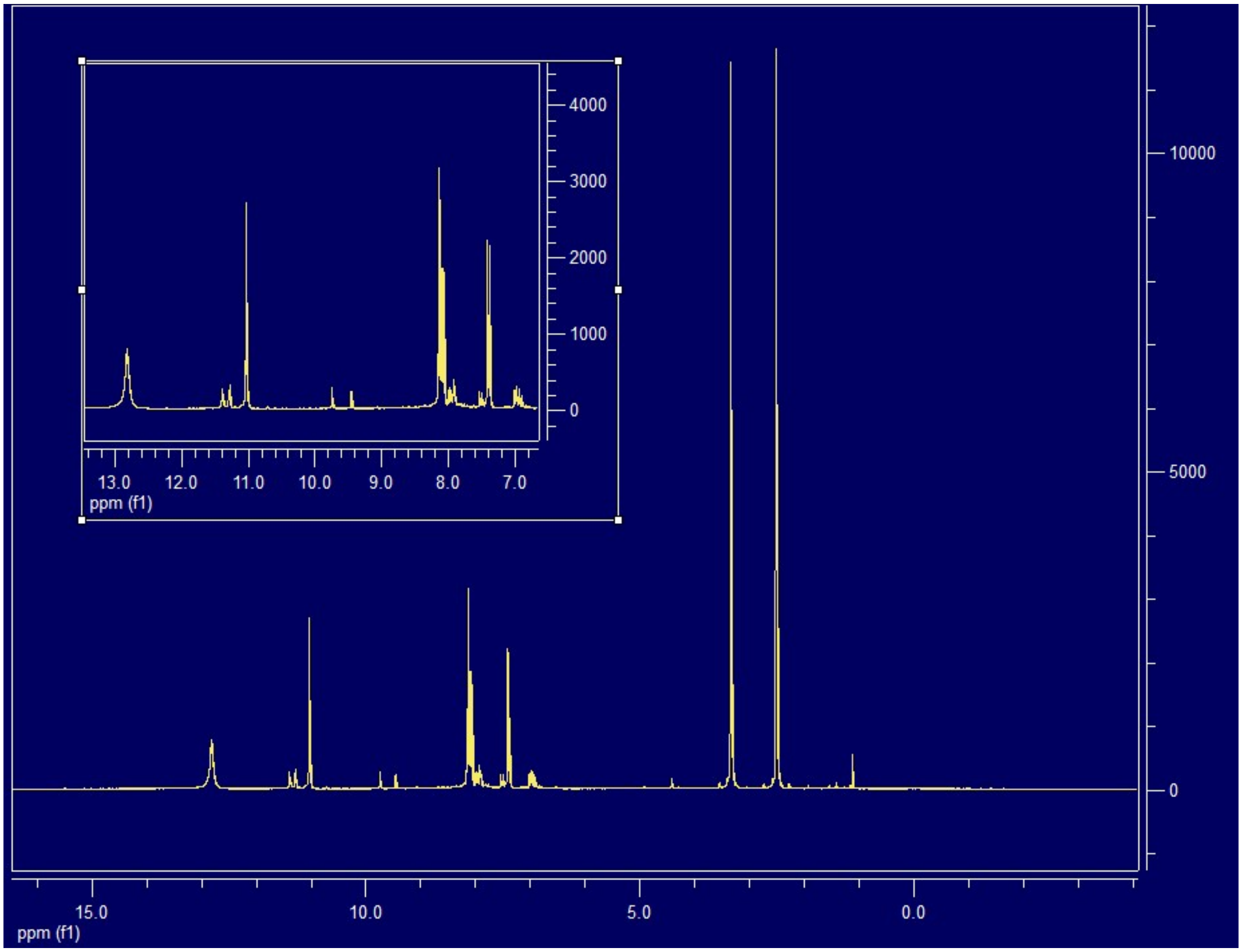

^{1}H NMR of **16**

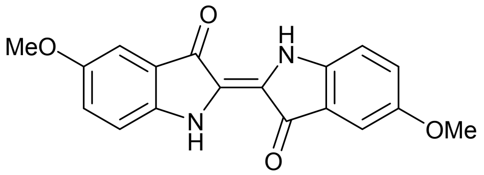

16

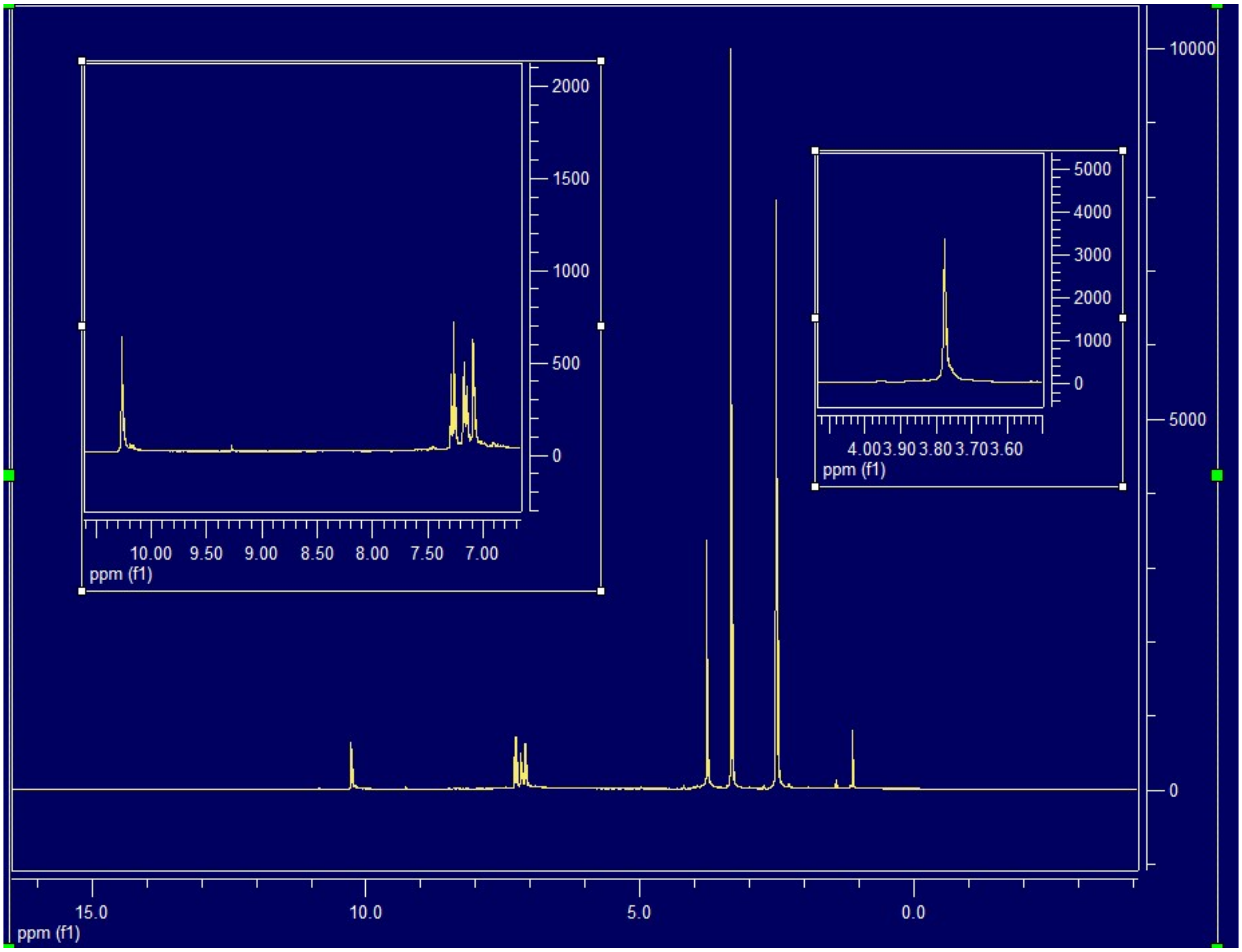

^{13}C NMR of **17**

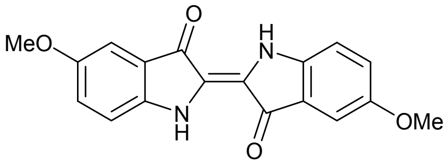

17

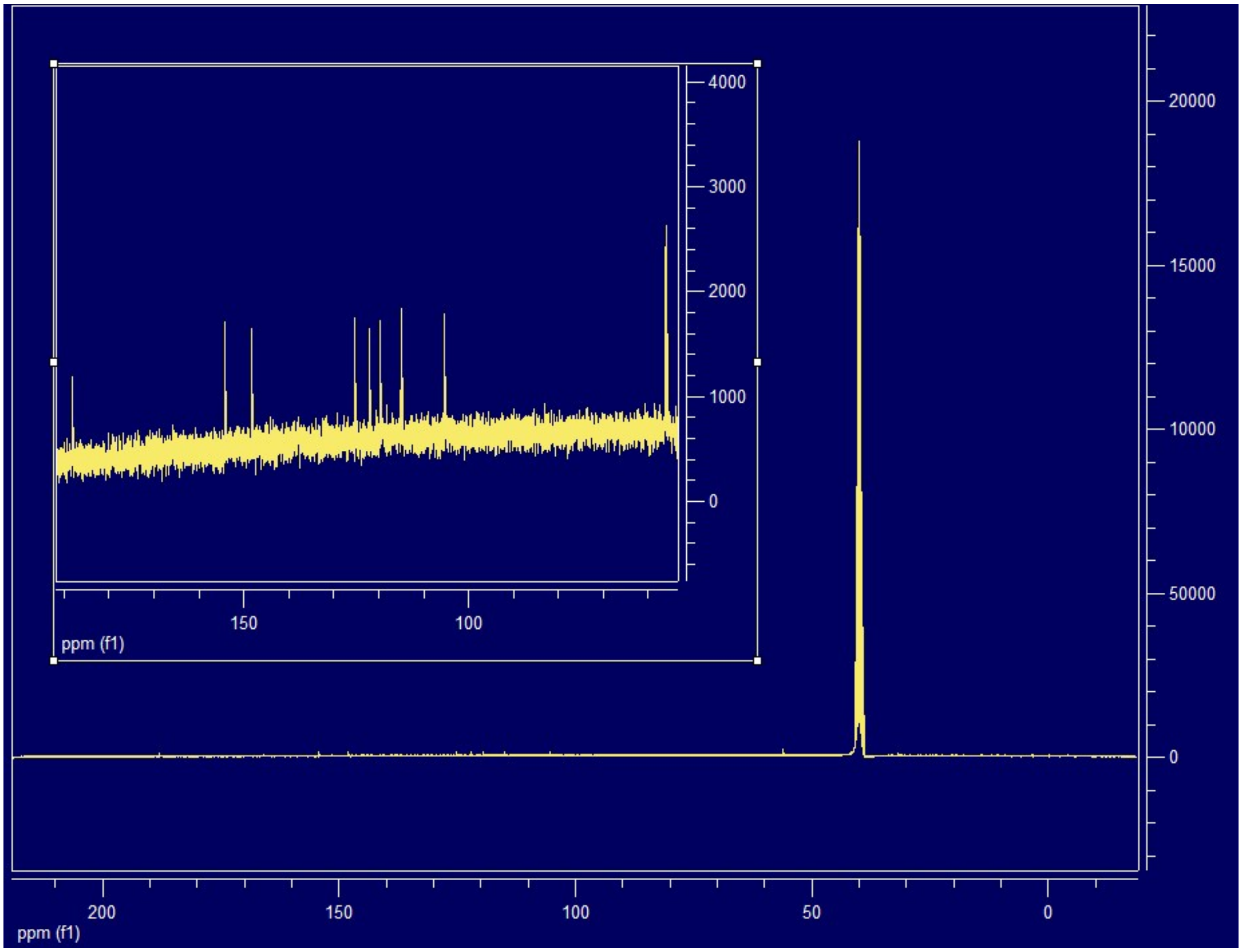

NMR of **15**.

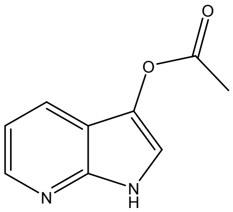

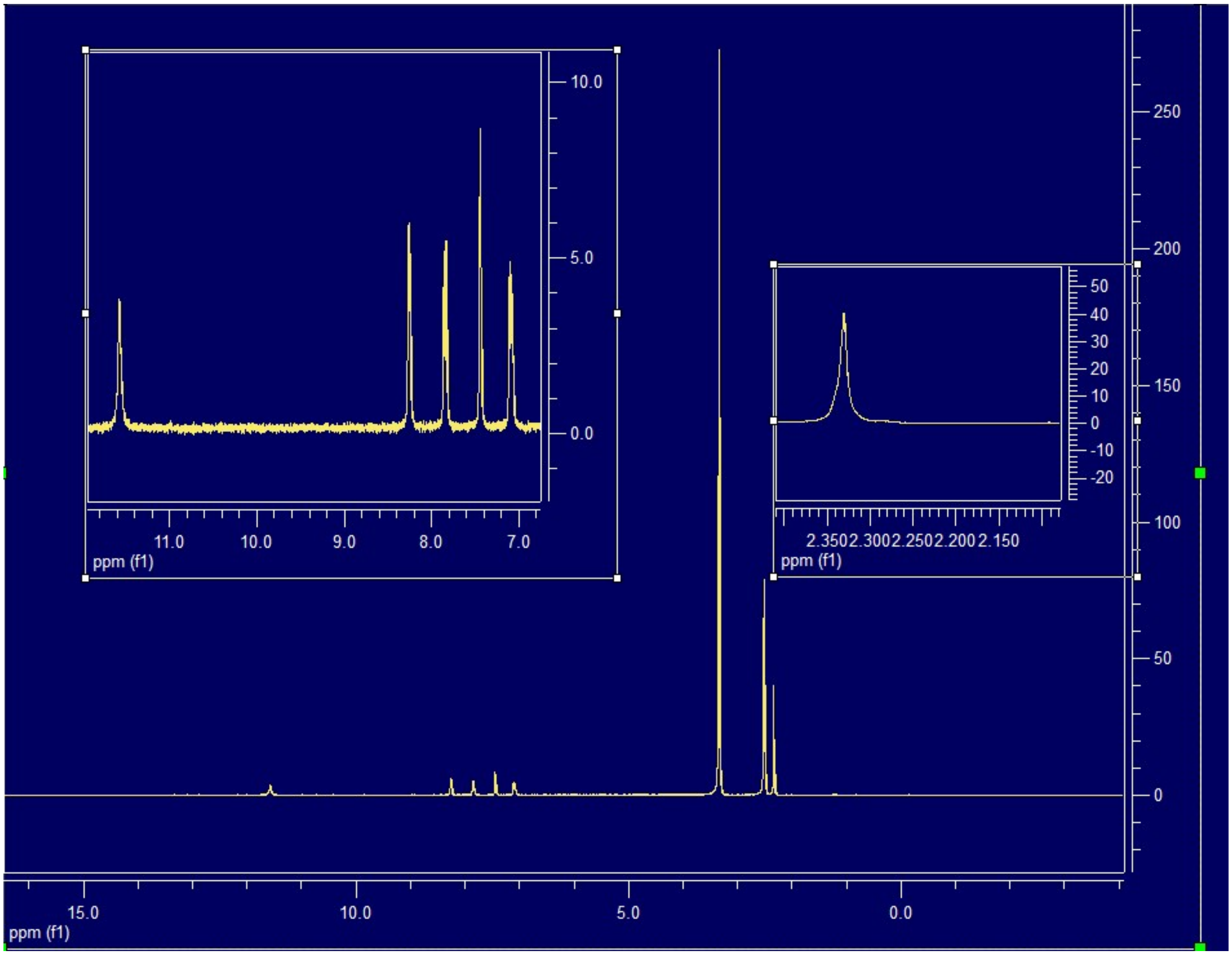

10.0
5.0
0.0
11.0
10.0
9.0
8.0
7.0
ppm (f1)
50
40
30
20
10
0
-10
-20
2.350
2.300
2.250
2.200
2.150
ppm (f1)
250
200
150
100
50
0
15.0
10.0
5.0
0.0
ppm (f1)

4. References

[1]X. Cheng, K-H. Merz, S. Vatter, J. Christ, S. Wölfl, *Bioorg. Med. Chem*., 2014, **22**, 247.

[2]N. M. Cuong, B. H. Tai, D. H. Hoan, *Nat. Prod. Res*. 2010, **24**, 99.

[3] http://www.biosite.dk/leksikon/indigo.htm

[4] Z-L. Wu, P. Aryal, O. Lozach, Meijer, L.; Guengerich, F. P. *Chem. Biodiversity,* 2005, **2**, 51.

[5] X. Cheng, K-H. Merz, S. Vatter, J. Zeller, S. Muehlbeyer, A. Thommet, J. Christ, S. Wölfl, G. Eisenbrand, *J. Med. Chem.* 2017, **60**, 4949.

[6] M. Saduaskas, R. Statkeviĉiũté, J. Vaitenkũnas, V. Petkeviĉius, V. Ĉasaité, R. Gasparraviĉiũté, R. Meškys, *Dyes Pigments,* 2020, **173**, 1.

[7] (a) P. Friedländer, S. Bruckner, G. Deutsch *Liebigs Annalen*, 2012, **388**, 23. (b) P.W. Sadler, *J. Org. Chem*., 1956, 21, 316.

[8] K. Lie, P. Wen, J. Lie, G. Huang, *Synthesis*, 2010, **21**, 3623.
